# The Catgut Ligature

**Published:** 1917-09-22

**Authors:** 


### THE CATGUT LIGATURE.

### A Technique for its Use in Civil Practice.

The writer had the privilege of being one of the late Lord Lister's senior students in the natal, and even in the prenatal, days of the catgut ligature; and has followed its development with keen interest. From the first Lord Lister's idea was to substitute an absorbable ligature for the silk threads which he had already been able to render safe as buried sutures. Two points were present in his mind and teaching. The first was that the catgut should be strong enough to stand the strain of cutting through the middle and inner coats of the artery and retaining its firm hold upon the outer coat until the process of healing should have sealed the vessel. The second was that the material used for antiseptic reasons should not be sufficiently caustic to produce necrosis. It is needless to follow the ever-changing detail by which this was secured—the iodine process, for most purposes, displaced all others.

### Two Forms of Iodised Catgut.

There are two forms of iodised catgut in use. In the older one the catgut is sterilised by iodine spirit solution, and then the superfluous iodine is washed out by the spirit in which the sterile ligature is preserved for use. In the more recent process the iodine is used in watery solution with potassium iodide, and the catgut remains in it until used. The objection to the older process is that the catgut is less resistent in making a firm knot, and less reliable in the duration of its hold. This somewhat limits its usefulness to the ligature of smaller vessels, and surface stitches. It is urged against the watery solution that it produces more local irritation and sets up some irritant inflammation, which is, of course, aseptic and self-terminable. There is some advantage in this when it is used to secure the stem of the appendix or for a suture of the peritoneum, in which cases the effusion of a little lymph only makes the scar more firm. A slight rise of the body temperature has been noted after its use, but no necrosis of tissue.

### The Development of Certain Principles.

When it is deemed prudent to limit the irritant action of the iodine, washing the ligature in sterile water will suffice. But Lister, in his earlier teaching, aimed at the healing of wounds under an aseptic scab. He did not accept without some doubt the late Sir James Paget's distinction between this and union by first intention. Cleansing, antiseptic and otherwise, rendered a wound safe under a scab if it brought the infection down to a degree which could be dealt with by the protective provisions of the tissues. In his earlier work he relied, after efficient cleansing, upon the use of a carbolised putty. A large surface was covered with this putty of various strengths, and the results were good. It fell to the writer's lot to point out that the carbolic content of the putty, even in its strongest form, was much below what he recognised as the lowest reliable antiseptic strength. He repeated the calculation, and said, "There must be some other power at work." He soon named that power as the control of movement which the firmness and clinging of the putty secured. The shellac dressing, which lacked this control, had a short life, and was displaced by the gauze, which formed a firmer support and ensured an aseptic scab. The deep stitches of silkworm gut, the buried stitches in the muscular and other tissues, were developments of the same principle, which reached its height in the sealing of the skin wound with gauze made adherent by collodion. The detachment of this adherent gauze need not give trouble if it is soaked with a solution of sulphuric ether in two parts of rectified spirit; but it is well not to disturb the skin stitches at the same dressing. A touch with ether soap removes any loose scab, and then the wound should be covered with a piece of sterile lint spread with an antiseptic ointment made with lard in place of vaseline. Duncan and Flockhart's antiseptic ointment is the one which the writer uses.

### The Process of Healing.

The principle of rest is secured by leaving the collodion scab untouched under the cotton pad for five days. Then the cotton pad is removed and the adherent gauze is touched with a weak iodine solution and the pad is replaced. On the tenth day the gauze is removed by the help of the ether solution, the silkworm gut stitches are removed and the ointment dressing applied. Two days' rest after this permits the aseptic lard to act as a skin food to the recent scar, while at the same time the lard soaks into the remnants of the catgut stitches and fits them for removal. On the twelfth day the lint is lifted from off the wound and a little ether soap is poured upon the surface of the skin. A light pad of cotton wool moistened with water is then used, after the manner of a shaving-brush, to work up a light lather, and as this is washed off the scar will be found free from all remnants of the catgut stitches and soundly healed.

### One of Lister's Principles Fulfilled.

There is little that is new in this detail beyond the later dealing with the catgut stitches, but those who follow the method will recognise that it fulfils one of the principles insisted upon by the master—that you may spend much of your life less usefully than in cultivating a gentle touch.

